# Pneumococcal transmission is driven by TNFR2+ regulatory T-cells

**DOI:** 10.3389/fimmu.2026.1840694

**Published:** 2026-07-20

**Authors:** Daan Beentjes, James Murray, Neil French, Caroline Vipond, Rong Xu, Aras Kadioglu

**Affiliations:** 1 Department of Clinical Infection, Microbiology and Immunology, University of Liverpool, Liverpool, United Kingdom; 2 Vaccines, Science Research & Innovation, Medicines and Healthcare Products Regulatory Agency, Potters Bar, United Kingdom

**Keywords:** carriage, pneumococcus (*Streptococcus pneumoniae*), regulatory T (Treg) cell, TNFR2, transmission

## Abstract

**Background:**

Pneumococcal serotype 1 (sequence type 217) is a leading cause of invasive disease outbreaks in Sub-Saharan Africa, causing significant morbidity and mortality. Understanding the transmission dynamics of hypervirulent strains such as ST217 is key to developing therapies and vaccines that can reduce outbreak incidence.

**Methods:**

The transmission dynamics of ST217 were investigated using an adolescent mouse pneumococcal transmission model, representing the population mainly affected by disease outbreaks in Sub-Saharan Africa.

**Results:**

We found that TNF receptor-2 (TNFR2)-positive regulatory T-cells (Tregs) paradoxically create the conditions that promote ST217 transmission. During ST217 colonisation, TNFR2^+^ Tregs accumulate in the nasopharynx and suppress IL-17A-producing γδT and Th17 cells, leading to reduced neutrophil-mediated pneumococcal clearance. This attenuated inflammatory response increases ST217 carriage density, consequently increasing nasal shedding to levels that increase transmission. Furthermore, we show that these TNFR2^+^ Treg responses are strain-dependent and driven by the pneumococcal toxin pneumolysin. The low-transmission serotype 23F exhibits low pneumolysin activity, resulting in impaired TNFR2^+^ Treg responses. This, in turn, enhances activation of the γδT17-neutrophil axis, which promotes pneumococcal clearance and disrupts 23F transmission.

**Conclusion:**

This study demonstrates an unexpected but key role of TNFR2^+^ Tregs in transmission of hypervirulent pneumococci.

## Introduction

*Streptococcus pneumoniae* (the pneumococcus) is the main cause of pneumonia and bacterial meningitis worldwide (1). Pneumococcal infections remain a major health burden, especially in low-income countries (2). Pneumococcal meningitis outbreaks continue to occur in Sub-Saharan Africa, mainly affecting (young) adults (3). Serotype 1 — predominantly sequence type (ST)217 — is responsible for most invasive disease cases in this region (3).

Asymptomatic nasopharyngeal carriage of *S. pneumoniae* is a prerequisite for invasive infection and plays a central role in host-to-host transmission (4). Transmission occurs through respiratory droplets and aerosols or contact with contaminated fomites (5). While transmission is a crucial stage in pneumococcal pathogenesis, the bacterial and host factors that contribute to transmission remain poorly understood. Host immunity during carriage is better characterised, with T-helper (Th)17 cells essential for clearing carriage and providing protection against colonisation through interleukin-17A (IL-17A) production, which is key for macrophage and neutrophil recruitment (6–8). γδT-cells, another principal source of IL-17A, are important for pathogen recognition, early immune activation, and mediate pneumococcal clearance (6, 9). Regulatory T-cells (Tregs) suppress Th17 and γδT-cell responses during bacterial infections (6, 8, 10). While Tregs minimise tissue damage by preventing excessive inflammation, they simultaneously contribute to the chronicity of infections by impairing bacterial clearance (6, 11, 12). We have previously demonstrated that Tregs maintain high carriage density in the nasopharynx and prolong carriage duration, suggesting a potential role in pneumococcal transmission as well (11).

Tumour Necrosis Factor (TNF) is important for Treg activation and stabilising Forkhead box P3 (Foxp3) — a master regulator of Treg development and function (13, 14). TNF is quickly upregulated upon pneumococcal colonisation and activates Tregs by binding to TNF receptor 2 (TNFR2), which is primarily found on Tregs (13–15). TNFR2^+^ Treg subsets display the highest suppressive activity (16). We recently showed that pneumococcus-exposed Tregs upregulate TNFR2 *in vivo*, and TNF-TNFR2 signalling stabilises Treg populations (6). However, the role of TNFR2 signalling in Treg-mediated control of pneumococcal carriage and its impact on transmission remains unknown.

Previous studies have shown that carriage dynamics and transmission rates are strain-dependent (17, 18). While some strains are predominantly associated with asymptomatic nasopharyngeal colonisation, others, such as ST217, possess a high invasive disease potential (19). Understanding the transmission dynamics of hypervirulent strains like ST217 is important for developing effective treatments and vaccines aimed at interrupting transmission and reducing outbreak incidence. Differences in pneumococcal-host immune interactions are likely to contribute significantly to the transmission potential of a strain, as the induction and evasion of immune responses are often strain-dependent (20, 21). Hence, the detailed study of host immunity during transmission is key to our fuller understanding of the transmission event. The aim of this study was therefore to elucidate these interactions using a host-to-host transmission model in adolescent mice, which reflects the age group most affected by outbreaks in Sub-Saharan Africa (3). Using this model, we compared the carriage and transmission dynamics of the outbreak-associated ST217 strain of serotype 1 with serotype 23F, which is rarely linked to invasive disease outbreaks, focusing on pneumococcal-host interactions and T-cell-mediated control of carriage and transmission.

## Methods

### Bacteria

Strains used in this study include serotypes 1 (B11510; ST217) and 23F (BVX11J) from adults attending antiretroviral therapy clinics in Blantyre’s Queen Elizabeth Central Hospital (Blantyre, Malawi) (22), and a pneumolysin (Ply)-KO strain of ST217. The ST217Δ*ply* mutant was generated via allelic replacement of the *ply* gene with the aphA3 cassette, which confers resistance to kanamycin. Transformants were selected on kanamycin-supplemented medium (250 µg/ml), and successful allelic exchange at the *ply* locus was verified by polymerase chain reaction and confirmed by Sanger sequencing (23). Bacteria were grown at 37 °C in Brain heart infusion broth [Neogen, Ayr, United Kingdom (UK)] with 20% (v/v) heat-inactivated foetal bovine serum (Sigma-Aldrich, Gillingham, UK) or anaerobically grown on blood agar base number 2 (Neogen) containing 5% (v/v) defibrinated horse blood (TCS bioscience, Buckingham, UK) and 1 μg/ml gentamicin (Sigma-Aldrich). Bacteria were stored at -80 °C and washed in phosphate-buffered saline [PBS (Gibco, Thermo Fisher Scientific, Loughborough, UK)] before use. Bacteria were identified as *S. pneumoniae* by optochin sensitivity [5μg disks (Oxoid, Basingstoke, UK)] and α-haemolysis on blood agar.

### Mice

Five-week-old female C57BL/6J (wild type) mice were purchased from Charles River (Margate, UK) and acclimatised for one week before infection. Male heterozygous Foxp3^DTR/EGFP^ DEpletion of REGulatory T-cells (DEREG) mice, generously provided by Tim Sparwasser (TWINCORE, Hannover), were mated with female C57BL/6J (WT) mice, to produce heterozygous Foxp3^DTR/EGFP^ and WT littermates. DEREG mice possess a diphtheria toxin receptor (DTR)-EGFP transgene under the control of an additional Foxp3 promoter, permitting specific depletion of Tregs by administration of diphtheria toxin (DT) (24). B6.129S2-Tnfrsf1btm1Mwm/J (TNFR2^−/−^) mice, which have a knockout of the TNF receptor superfamily 1b (*tnfrsf1b*) gene encoding TNFR2, were purchased from The Jackson Laboratory [Bar Harbor, Maine, United States (US)] and bred in-house to produce homozygous knockouts. TNFR2^-/-^ and WT mice were age-matched for experiments. Mice were housed at 22 °C ± 1 °C (40-65% humidity) in individually ventilated cages (12-hour light-dark cycle), with free access to water and food. All animal procedures were conducted under project license PP2832279, in accordance with the University of Liverpool’s Animal Welfare and Ethical Review Body, and in compliance with the UK Animals (Scientific Procedures) Act 1986.

### Mouse-to-mouse transmission model

Two six-week-old female mice (i.e., index mice) were lightly anesthetised (mixture of O_2_ and isoflurane) and intranasally infected with 10^5^ colony-forming units (CFUs) of *S. pneumoniae* in 10 µl as described previously (23). Index mice were co-housed with three uninfected mice (i.e., contact mice) for a total of 10 days. On day 3 post-infection (p.i), all mice were intranasally challenged with 10^3^ plaque-forming units (10 µl) of influenza A virus (IAV) (HKx31; H3N2) to facilitate nasal secretions (18). Pneumococcal shedding was quantified daily by gently pressing the nares of each mouse 20 times against a blood agar plate. This was performed under light anaesthesia to minimise stress in mice. On day 10 p.i., pneumococcal counts in the nasopharynx of contact mice were determined and the transmission rate, expressed as the percentage of colonised contact mice, was calculated.

### Pneumococcal carriage model

Female C57BL/6J mice were lightly anesthetised and intranasally infected with 1 x 10^5^ CFU of *S. pneumoniae* in 10 µl PBS to induce asymptomatic carriage (25). For Treg depletion in DEREG mice, DEREG and WT mice were intraperitoneally treated with DT (Sigma-Aldrich; 1µg in 100 µl PBS) on days 0 and 1 of carriage.

### Nasopharyngeal tissue processing

Nasopharyngeal tissue was obtained as described previously (25). Tissues were passed through a 40-micron EASY cell strainer (Greiner, Stonehouse, UK). A sample was taken for bacterial enumeration as described previously (25). Remaining cells were spun down (400 rcf; 5 min), cell supernatants were stored at -80 °C, and cells were washed in Hanks’ balanced salt solution (Sigma-Aldrich) before further processing.

### Flow cytometry

Nasopharyngeal single-cell suspensions were stained with Fixable Aqua LIVE/DEAD™ stain (Invitrogen, Thermo Fisher Scientific). Cells were washed in Dulbecco’s PBS (Gibco) and stained with antibodies listed in **Supplementary Table 1**. Samples were acquired using a BD FACSCanto™ II flow cytometer. FlowJo™ V10.8.0 (Ashland, US) was used for analysis. See **Supplementary Figure 1** for the gating strategy.

### ELISA

Cell supernatants of nasopharyngeal tissue were used for Enzyme-linked immunosorbent assays (ELISA) to quantify IL-17A (Invitrogen) and TNF (Invitrogen) levels. ELISAs were performed according to the manufacturer’s instructions.

### Haemolytic activity assay

Haemolytic activity was determined as described previously (26). Briefly, pneumococci were lysed with 0.1% (w/v) sodium deoxycholate (Sigma-Aldrich) and protein concentration was determined using a Pierce™ BCA Protein Assay kit (Thermo Fisher Scientific) according to manufacturer’s instructions. Lysates (5 µg protein) were diluted 1:1 with PBS containing 4% (v/v) red blood cells from defibrinated sheep blood (TCS bioscience) and incubated for 30 minutes at 37 °C. Cells were spun down and haemoglobin levels (OD_540_) were measured in supernatant.

### Pneumolysin gene sequencing

Genomic DNA was extracted from mid-log grown cultures of ST217 and 23F using DNeasy^®^ Blood & Tissue Kit (Qiagen, Manchester, UK) according to manufacturer’s instructions. Whole-genome sequencing was performed in-house at the Medicines and Healthcare products Regulatory Agency (MHRA, UK) using Oxford Nanopore long-read sequencing (Rapid Barcoding Kit SQK-RBK114.24; Oxford UK) and short-read sequencing. Barcoded libraries were pooled and sequenced on R10.4.1 Flow cells (FLO-MIN114). For short-read sequencing, libraries were prepared using the Illumina DNA PCR-Free Prep kit with Illumina DNA/RNA UD Indexes, Tagmentation (Illumina, USA), involving bead-linked transposome-mediated DNA fragmentation and adapter tagging followed by index ligation. Libraries were quantified using the KAPA Library Quantification Kit (Roche) on an AriaMX Real-Time PCR System (Agilent Technologies, USA), pooled, and sequenced on a NextSeq 2000 platform using a NextSeq P1 300-cycle XLEAP kit with 151 bp paired-end reads (Illumina, USA). Assembled genomes were annotated using Prokka. Analysis was performed in a Windows Subsystem for Linux (Ubuntu) environment. The *ply* gene was identified from Prokka annotation files and extracted from genome assemblies using SAMtools (v1.22.1). Nucleotide sequences were aligned using MAFFT (v7.525), pairwise similarity was assessed using BLASTN (NCBI BLAST+ v2.16.0), and single nucleotide polymorphisms were identified using MUMmer (v3.1). Predicted amino acid sequences generated with Transeq (EMBOSS v6.6.0.0) were aligned using MAFFT to identify amino acid substitutions. Protein structures were predicted using AlphaFold3 (2026.04.16) and visualised in PyMOL (v3.0.3).

### Statistics

GraphPad Prism version 8.4.3 (San Diego, US) was used to perform statistical analysis. All statistical analyses were performed using two-tailed tests. Data show the mean ± the standard error of the mean (SEM). An adjusted p-value lower than 0.05 was considered statistically significant.

## Results

### Transmission of hypervirulent serotype 1 is dependent on TNFR2 signalling

**Figure 1:**
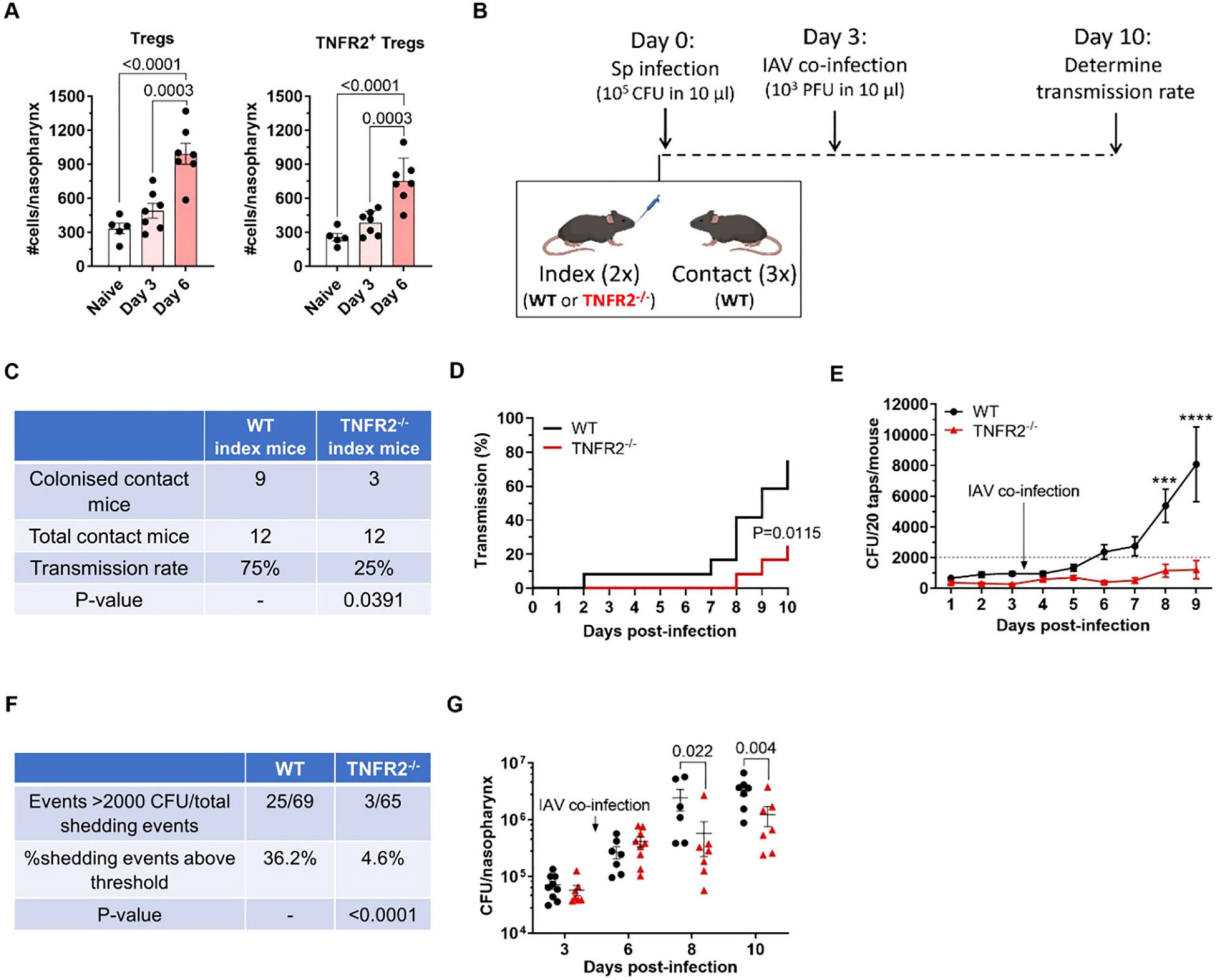
TNFR2-signalling enhances pneumococcal transmission. **(A)** Number of (TNFR2^+^) Tregs (CD3^+^CD4^+^Foxp3^+^) in the nasopharynx on days 3 (n=7) and 6 (n=7) post-infection. Naïve mice (n=5) acted as a control. Data shown are from two independent experiments and was analysed with ordinary one-way ANOVA with Holm-Sidak’s multiple comparisons test. **(B)** Experimental timeline of IAV co-infection transmission model. Created in BioRender. Beentjes, D. (2026) https://BioRender.com/intdybk. Two female wildtype or TNFR2^-/-^ C57BL/6J ‘index’ mice were intranasally infected with a carriage dose of pneumococcal serotype 1 (ST217) and co-housed for 10 days with three uninfected ‘contact’ mice. On day 3 post-infection, all mice were intranasally infected with IAV. **(C)** Number of colonised contact mice and transmission rates when using WT or TNFR2^-/-^ mice as index mice. Transmission was monitored in four cages per group in four independent experiments. **(D)** Transmission of ST217 over time in WT and TNFR2^-/-^ mice. Data shown are the percentages of colonised contact mice (n=12) per day. Contact mice were considered colonised if pneumococci could be recovered on two consecutive days by nose tapping. **(E)** Daily average shedding levels of ST217 (CFU/20 nose taps/mice [± SEM]) in WT and TNFR2^-/-^ mice (n=8 per group). Dashed line shows the transmission threshold of 2000 CFU/20 nose taps. **(F)** Percentage of shedding events in WT and TNFR2^-/-^ that exceed the transmission threshold of 2000 CFU/20 nose taps. **(G)** Pneumococcal density (± SEM) in the nasopharynx of WT and TNFR2^-/-^ mice on days 3 (n=9; n=7), 6 (n=7; n=9), 8 (n=6; n=7), and 10 (n=7; n=7) post-infection. A Fisher’s exact test was used to compare transmission rates and the fraction of shedding events that exceed the transmission threshold **(C, F)**. Transmission rates over time were compared using Mantel-Cox analysis **(D)**. Carriage densities and shedding levels were compared with Multiple t-tests (corrected with Holm-Sidak method) **(E, G)**. ***P<0.001. ****P<0.0001. Sp, *Streptococcus pneumoniae*. IAV, Influenza A virus.

We have previously shown that Tregs are activated during pulmonary pneumococcal infection, a process dependent on TNF-TNFR2 signalling (6). Interestingly, we found that both total Tregs and TNFR2^+^ Tregs accumulate in the nasopharynx of colonised mice during carriage of serotype 1 (ST217) (**Figure 1A**). To investigate their role in carriage and transmission, we used our recently developed adolescent mouse IAV co-infection transmission model (**Figure 1B**). ST217 is highly transmissible in WT mice (75% transmission rate) (**Figure 1C**). However, its transmission rate was significantly reduced when using TNFR2^-/-^ mice as index mice (25%; P = 0.0391). To further explore the transmission dynamics of ST217 in WT and TNFR2^-/-^ mice, we monitored transmission over time by collecting nasal secretions from contact mice through daily nose tapping (**Figure 1D**). Contact mice were considered colonised when pneumococci could be recovered from nasal secretions on two consecutive days. While transmission occurred as early as day 2 in WT mice, transmission was delayed until day 8 p.i. when using TNFR2^-/-^ mice as index mice (**Figure 1D**). Mantel-Cox analysis showed that transmission was significantly impaired when using TNFR2^-/-^ mice (P = 0.0115).

The number of pneumococci shed by a carrier influences transmission potential (27). We therefore measured ST217 shedding levels in WT and TNFR2^-/-^ mice. Shedding in WT mice increased from day 6, peaking on days 8 and 9 p.i., while TNFR2^-/-^ mice maintained low shedding levels (**Figure 1E**). Previous studies demonstrated that shedding must exceed a threshold level to permit transmission (27). In WT mice, most contact mice acquired *S. pneumoniae* from day 7 p.i., when shedding levels exceeded ~2000 CFUs (**Figure 1E**). We therefore established a transmission threshold of 2000 CFUs. 36.2% of shedding events in WT mice exceeded this threshold, compared to only 4.6% in TNFR2^-/-^ mice (**Figure 1F**), highlighting that TNFR2 signalling enhances shedding to levels that permit transmission.

We next investigated whether the low shedding in TNFR2^-/-^ mice was due to reduced pneumococcal carriage density. Carriage densities were similar between groups until day 6 p.i. (**Figure 1G**). However, from day 8 p.i., carriage density in WT mice increased to significantly higher levels compared to TNFR2^-/-^ mice, correlating with the rise in shedding and transmission observed in these animals (**Figures 1D, E**).

IAV co-infection significantly impacts pneumococcal carriage dynamics, an interaction that varies with pneumococcal strain and is affected by several host factors (28, 29). To confirm that TNFR2-mediated control of pneumococcal colonisation and shedding is independent of IAV co-infection, we compared shedding levels and carriage densities between WT and TNFR2^-/-^ mice in the absence of IAV. Even without IAV co-infection, WT mice showed significantly higher shedding levels and pneumococcal densities (**Supplementary Figure 2**).

Collectively, our results indicate that TNFR2 signalling significantly contributes to shedding and transmission of ST217 by maintaining high carriage density. Increased carriage density leads to enhanced shedding levels, which drives transmission.

### TNFR2 signalling suppresses IL-17A-mediated recruitment of innate immune cells during carriage

**Figure 2:**
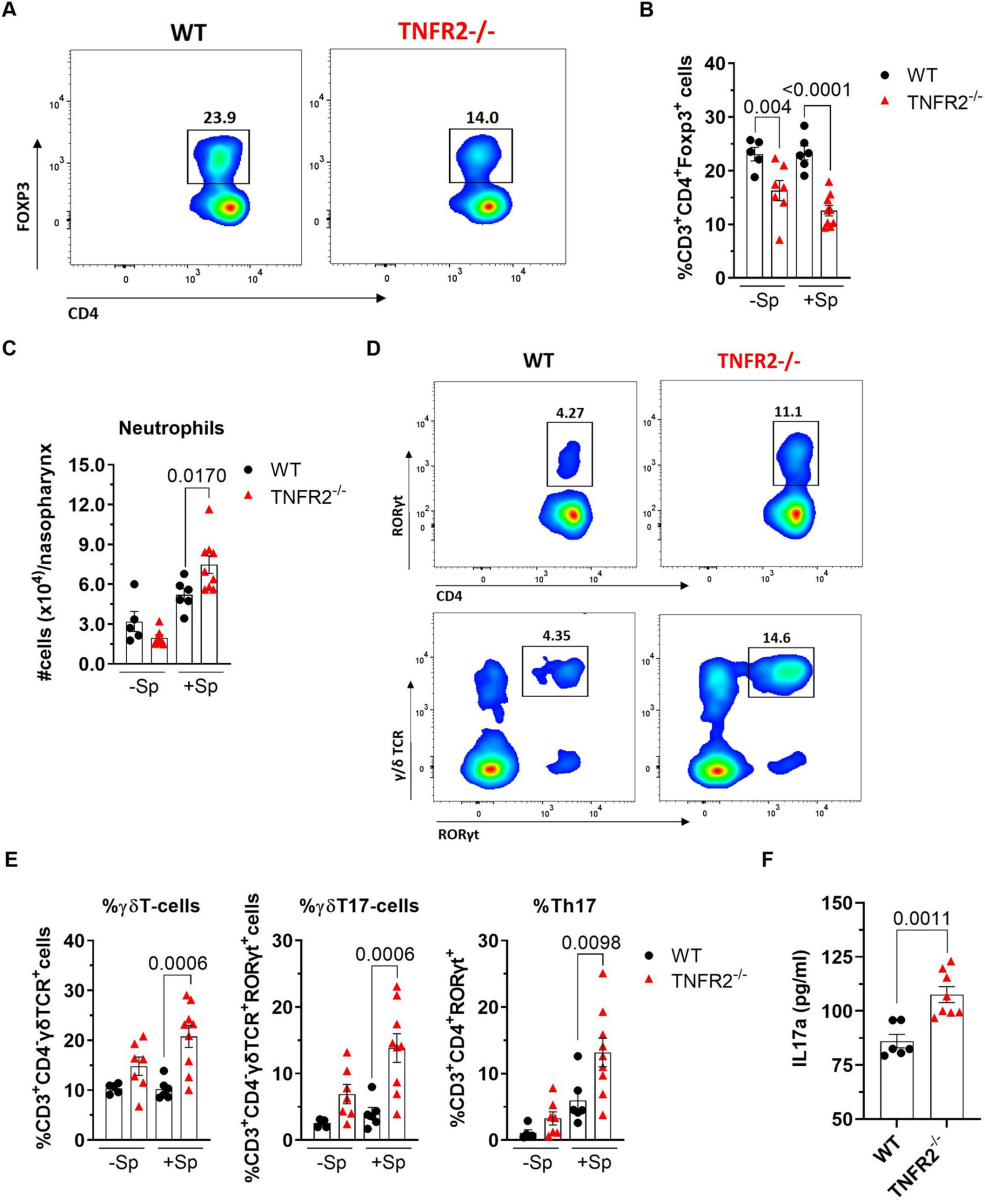
TNFR2^-/-^ index mice display increased IL17-mediated inflammation. **(A–F)** WT and TNFR2^-/-^ C57BL/6J mice were intranasally infected with ST217 and then co-infected with IAV. Immune responses in the nasopharynx of co-infected WT (n=6) and TNFR2^-/-^ mice (n=9) (+Sp) were determined on day 6 post-infection using flow cytometry. WT (n=5) and TNFR2^-/-^ (n=7) mice infected with IAV-only served as controls (-Sp). Data shown are from three independent experiments. **(A)** Representative flow cytometry plots of the proportion of Tregs (CD3^+^CD4^+^Foxp3^+^) in the nasopharynx of WT and TNFR2^-/-^ mice. **(B)** Treg proportions in the nasopharynx of WT and TNFR2^-/-^ mice. **(C)** Neutrophil (Ly6G^+^CD11b^+^) numbers in the nasopharynx of WT and TNFR2^-/-^ mice. **(D)** Representative flow cytometry plots of the proportion of Th17 cells (CD3^+^CD4^+^RORγt^+^) and γδT17-cells (CD3^+^CD4^-^γδTCR^+^RORγt^+^) in the nasopharynx of WT and TNFR2^-/-^ mice. **(E)** Proportion of γδT-cells (CD3^+^CD4^-^γδTCR^+^), γδT17-cells, and Th17 cells in the nasopharynx of WT and TNFR2^-/-^ mice. **(F)** IL-17A levels in nasopharyngeal cell supernatant of WT (n=6) and TNFR2^-/-^ (n=8) mice. Immune cell responses between groups were compared with ordinary one-way ANOVA with Holm-Sidak’s multiple comparisons test. Cytokine data were analysed with unpaired t-test. Sp, *Streptococcus pneumoniae*; IAV, Influenza A virus.

Having shown the key involvement of TNFR2 signalling in ST217 transmission, we compared Treg responses between WT and TNFR2^-/-^ mice during carriage. Consistent with our previous finding that TNFR2 signalling stabilises Treg populations in the lungs during pneumococcal pneumonia (6), we observed significantly lower Treg frequencies in the nasopharynx of TNFR2^-/-^ mice compared to WT mice on day 6 p.i., indicating that TNFR2 signalling is also crucial for maintaining Treg populations in the nasopharynx during carriage (**Figures 2A, B**).

IL-17A-mediated inflammation, which includes the recruitment of neutrophils and macrophages, is essential for clearing pneumococcal colonisation (7). We assessed the effect of Treg reduction in the nasopharynx by measuring IL-17A-producing γδT (γδT17) (CD3^+^, CD4^-^, γδTCR^+^, RORγt^+^) and Th17 (CD3^+^, CD4^+^, RORγt^+^) cells, as well as neutrophil (CD11b^+^, Ly6G^+^) recruitment in WT and TNFR2^-/-^ mice on day 6 p.i. TNFR2^-/-^ mice showed increased numbers and frequencies of all cell types in the nasopharynx and elevated IL-17A levels in nasopharyngeal cell supernatants compared to WT controls (**Figures 2C–F**). This increased immune response was not due to differences in bacterial density as the pneumococcal load on day 6 p.i. was similar between TNFR2^-/-^ and WT mice (**Figure 1G**).

In the absence of IAV, TNFR2^-/-^ mice also showed lower Treg frequencies and increased γδT17 and Th17 cells in the nasopharynx (**Supplementary Figure 3**). Additionally, a significantly higher number of macrophages (CD11b^+^, F4/80^+^) was measured in TNFR2^-/-^ mice compared to WT mice (**Supplementary Figure 3**). A trend towards increased neutrophil recruitment was also observed, though not statistically significant (**Supplementary Figure 3**).

These results indicate that TNFR2 signalling is important for maintaining Treg populations in the nasopharynx, which suppress IL-17A-producing γδT-cells and Th17 cells during pneumococcal carriage. This leads to impaired neutrophil and macrophage responses, allowing high pneumococcal carriage density of ST217 and enhancing shedding and transmission.

### Treg depletion enhances IL-17A-mediated neutrophil recruitment, leading to reduced shedding during early carriage

**Figure 3:**
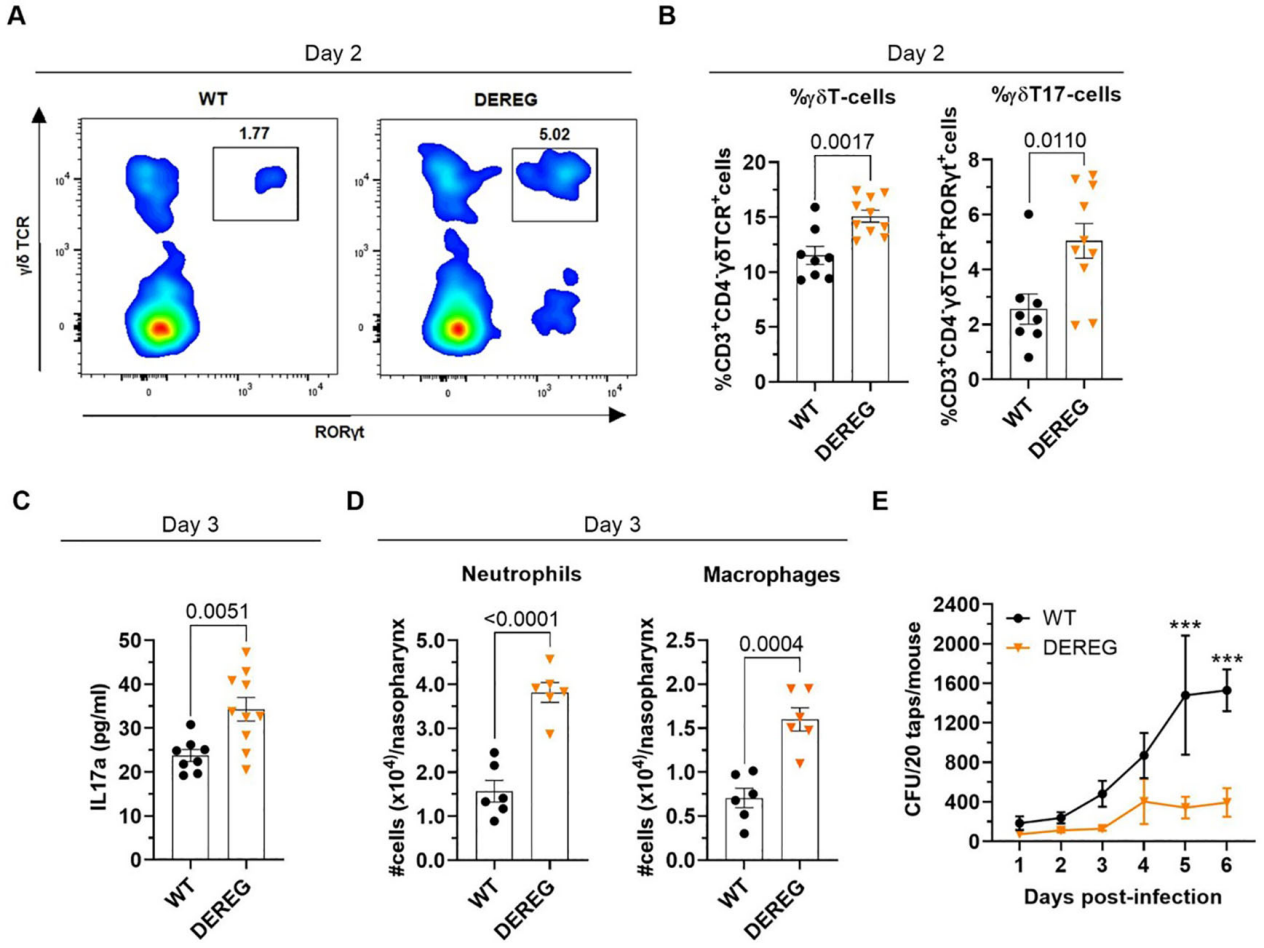
Treg-depleted mice display increased inflammation and reduced pneumococcal shedding during early carriage. **(A)** Representative flow plots of the frequency of γδT17-cells (CD3^+^CD4^-^γδTCR^+^RORγt^+^) in WT and DEREG mice. **(B)** Frequency of γδT-cells and γδT17-cells in WT (n=8) and DEREG (n=10) mice on day 2 post-infection. Data are from three independent experiments **(C)** IL-17A levels in nasopharyngeal cell supernatant of WT (n=8) and DEREG (n=10) mice on day 3 post-infection. Data shown are from three independent experiments and were analysed with an unpaired t-test. **(D)** Number of neutrophils and macrophages in WT (n=6) and DEREG (n=6) mice on day 3 post-infection. Groups were compared with unpaired t-tests. Data are from two independent experiments. **(E)** Daily average pneumococcal shedding levels (CFU/20 nose taps/mice [± SEM]) of ST217 in WT (n=6) and DEREG (n=7) mice. Data shown is from two independent experiments. Shedding levels between groups were analysed with multiple t-tests (corrected with Holm-Sidak method). ***P<0.001.

We have shown that TNFR2^-/-^ mice, which lack TNFR2^+^ Tregs, display increased IL-17A-mediated pneumococcal clearance. While TNF-TNFR2 signalling primarily affects Treg cell activation, it may influence other TNFR2-expressing immune cells (30, 31). To confirm the role of Tregs in controlling pneumococcal carriage and shedding, we used DEREG mice. DEREG mice possess a diphtheria toxin receptor (DTR) under the control of an additional Foxp3 promoter, permitting specific depletion of Tregs by administration of DT (24). DT treatment leads to over 95% depletion of Tregs in the nasopharynx of DEREG mice, while Treg populations in WT mice remain unaffected (**Supplementary Figure 4**). As expected, we measured increased proportions of γδT17-cells, elevated IL-17A levels, and significantly higher numbers of neutrophils, and macrophages in DEREG mice during early carriage (**Figures 3A–D**). DEREG mice also displayed more than a 2-fold reduction in shedding levels compared to WT littermates, with significant differences observed on days 5–6 p.i. (P = 0.0009; P = 0.0009) (**Figure 3E**). These results indicate that Tregs are crucial for suppressing IL-17A-mediated inflammation during early infection, leading to enhanced shedding levels.

### Low-transmission strain 23F is less capable of activating TNFR2^+^ Treg responses

**Figure 4:**
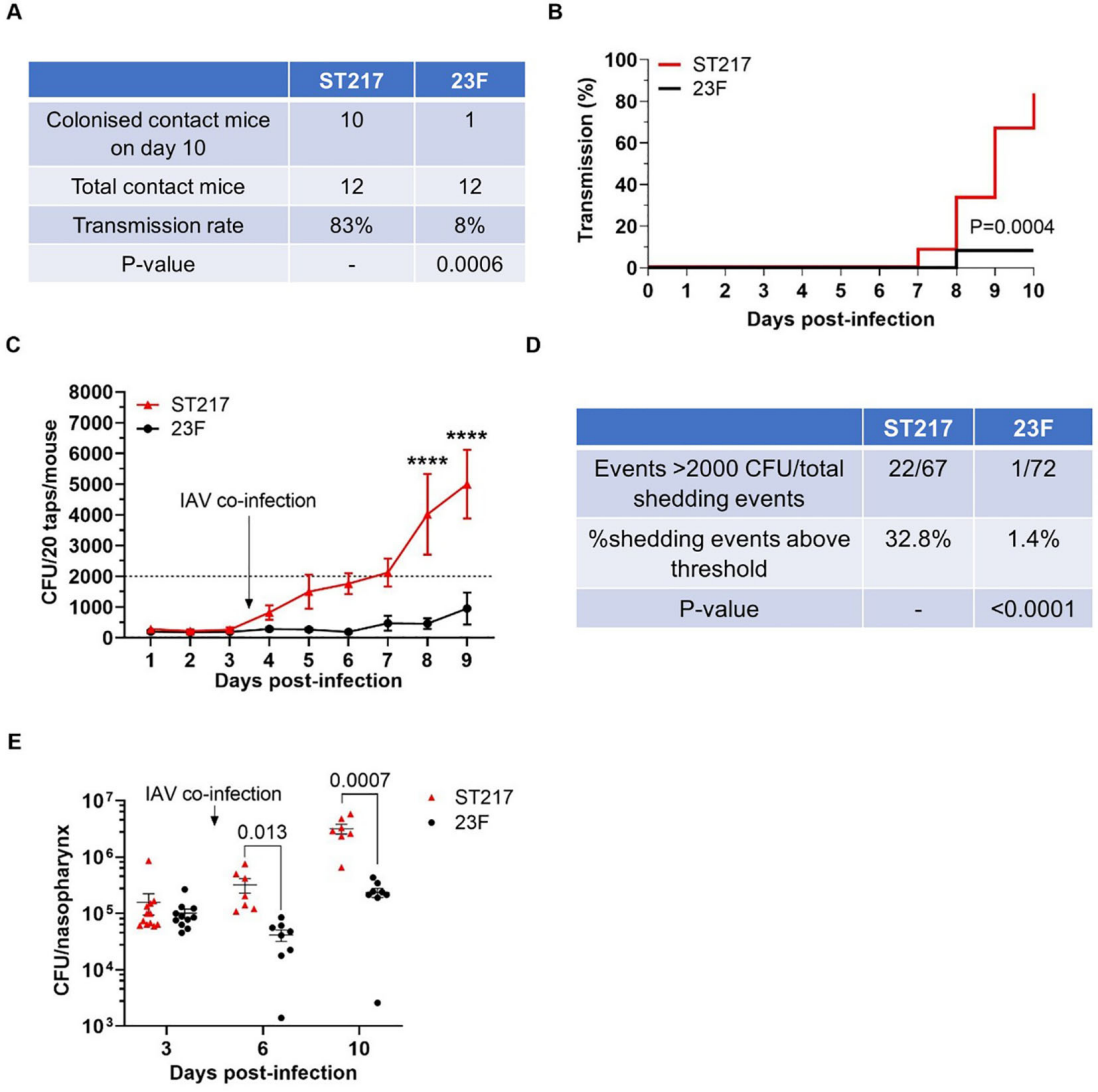
Serotype 1 (ST217) displays a higher transmission rate and higher shedding levels compared to serotype 23F. **(A)** Number of colonised contact mice and transmission rate (%) on day 10 post-infection. Transmission was monitored in 4 cages (n=12 contact mice) in two independent experiments. Transmission rates were compared with a Fisher’s exact test. **(B)** Transmission rate over time of ST217 and 23F. Data shown are the percentages of colonised contact mice (n=12) per day. Contact mice were considered colonised if pneumococci could be recovered on two consecutive days by nose tapping. Data were analysed with a Mantel-Cox test. **(C)** Daily average pneumococcal shedding levels of ST217 and 23F (CFU/20 nose taps/mice [± SEM]) (n=8 per group). Dashed line shows the transmission threshold of 2000 CFU/20 nose taps. Data shown are from two independent experiments. **(D)** Percentage of shedding events that exceed the transmission threshold of 2000 CFU/20 nose taps. Data were analysed with a Fisher’s exact test. **(E)** Pneumococcal density in the nasopharynx of ST217- and 23F-infected mice on days 3 (n=12; n=11), 6 (n=7; n=8), and 10 (n=7; n=8) post-infection. Data are from three independent experiments. Shedding levels and pneumococcal densities between strains were compared with multiple t-tests (corrected with Holm-Sidak method). ****P<0.0001. IAV, Influenza A virus.

Having established that (TNFR2^+^) Tregs play a key role in ST217 carriage and transmission, we investigated whether variations in Treg responses could explain strain-dependent transmission rates. We compared the transmission dynamics of ST217 with the low-transmission serotype 23F, which has a significantly lower transmission rate (8%) compared to ST217 (83%) (P = 0.0006) (**Figure 4A**). Transmission of 23F was delayed (day 8 vs day 7 p.i.) and significantly reduced compared to ST217 (P = 0.0004) (**Figure 4B**). Shedding of 23F was also markedly lower than ST217, both in the presence and absence of IAV (**Figure 4C**; **Supplementary Figure 5**). While 32.8% of the shedding events were measured above the transmission threshold for ST217, only 1.4% of events exceeded the threshold for 23F, indicating that 23F shedding levels were far from sufficient to allow transmission (**Figure 4D**). Consistent with our results, ST217 also carries at a higher density than 23F, regardless of IAV co-infection (**Figure 4E**; **Supplementary Figure 5**).

**Figure 5:**
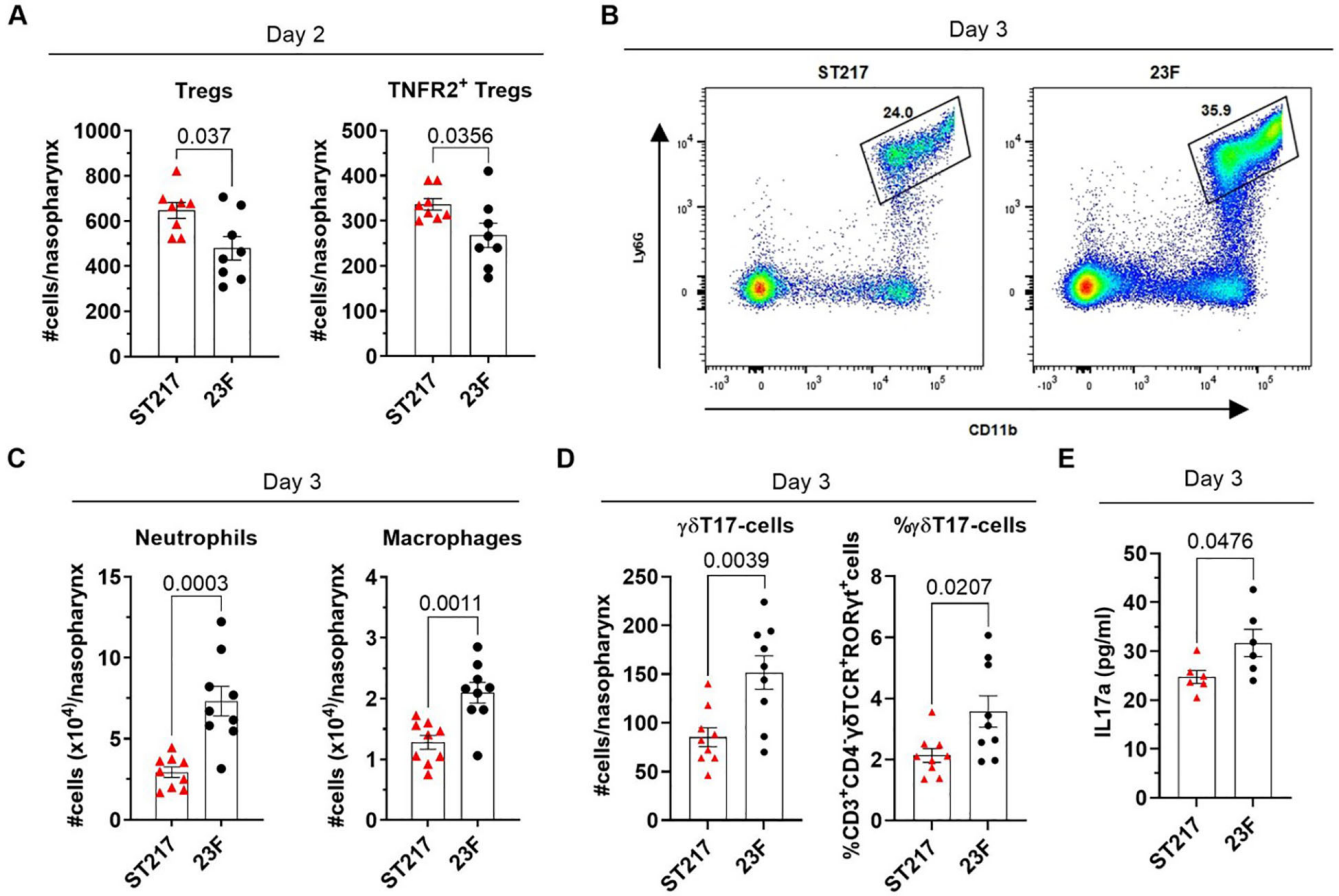
23F-infected mice show enhanced inflammation during early carriage **(A–E)** Female C57BL/6J mice were intranasally infected with a carriage dose of ST217 or 23F. Flow cytometry was used to determine immune responses in the nasopharynx. Data shown are from three independent experiments. **(A)** Number of (TNFR2^+^) Tregs (CD3^+^CD4^+^Foxp3^+^) in the nasopharynx of mice infected with ST217 (n=8) and 23F (n=8) on day 2 post-infection. Groups were compared with unpaired t-tests. **(B)** Representative flow cytometry plots of the proportion of neutrophils (Ly6G^+^CD11b^+^) in the nasopharynx of mice infected with ST217 and 23F on day 3 post-infection. **(C)** Number of neutrophils and macrophages (F4/80^+^CD11b^+^) in the nasopharynx of ST217- and 23F-infected mice (n=9 per group) on day 3 post-infection. **(D)** Number and frequency of γδT17-cells (CD3^+^CD4^-^γδTCR^+^RORγt^+^) in the nasopharynx of mice infected with ST217 and 23F on day 3 post-infection. **(E)** IL-17A levels in nasopharyngeal cell supernatant of ST217- and 23F-infected mice on day 3 post-infection (n=6 per group).

We then sought to determine whether low colonisation density and shedding of 23F correlated with a lack of TNFR2^+^ Treg response. We found significantly lower (TNFR2^+^) Foxp3^+^ Tregs in the nasopharynx of 23F-infected mice compared to ST217-infected mice (**Figure 5A**). This higher number of TNFR2^+^ Tregs was due to an increase in total Tregs, as the proportion of TNFR2^+^ Tregs were similar between groups (**Supplementary Figure 6**). Differences were not due to higher carriage density, as pneumococcal loads were similar between ST217 and 23F at that timepoint (**Supplementary Figure 7**). The reduced Treg response in 23F-infected mice was followed by enhanced neutrophil and macrophage responses (**Figures 5B, C**), significantly higher γδT17-cells (**Figure 5D**), and elevated IL-17A levels on day 3 p.i. (**Figure 5E**).

These results suggest that Treg induction during early colonisation is strain-dependent. Colonisation with 23F leads to low TNFR2^+^ Treg responses, which activate IL-17A-producing γδT17-cells, neutrophils, and macrophages, thereby promoting bacterial clearance and reducing pneumococcal shedding and transmission of 23F.

#### Pneumolysin (Ply) is crucial for Treg activation and host-to-host transmission

**Figure 6:**
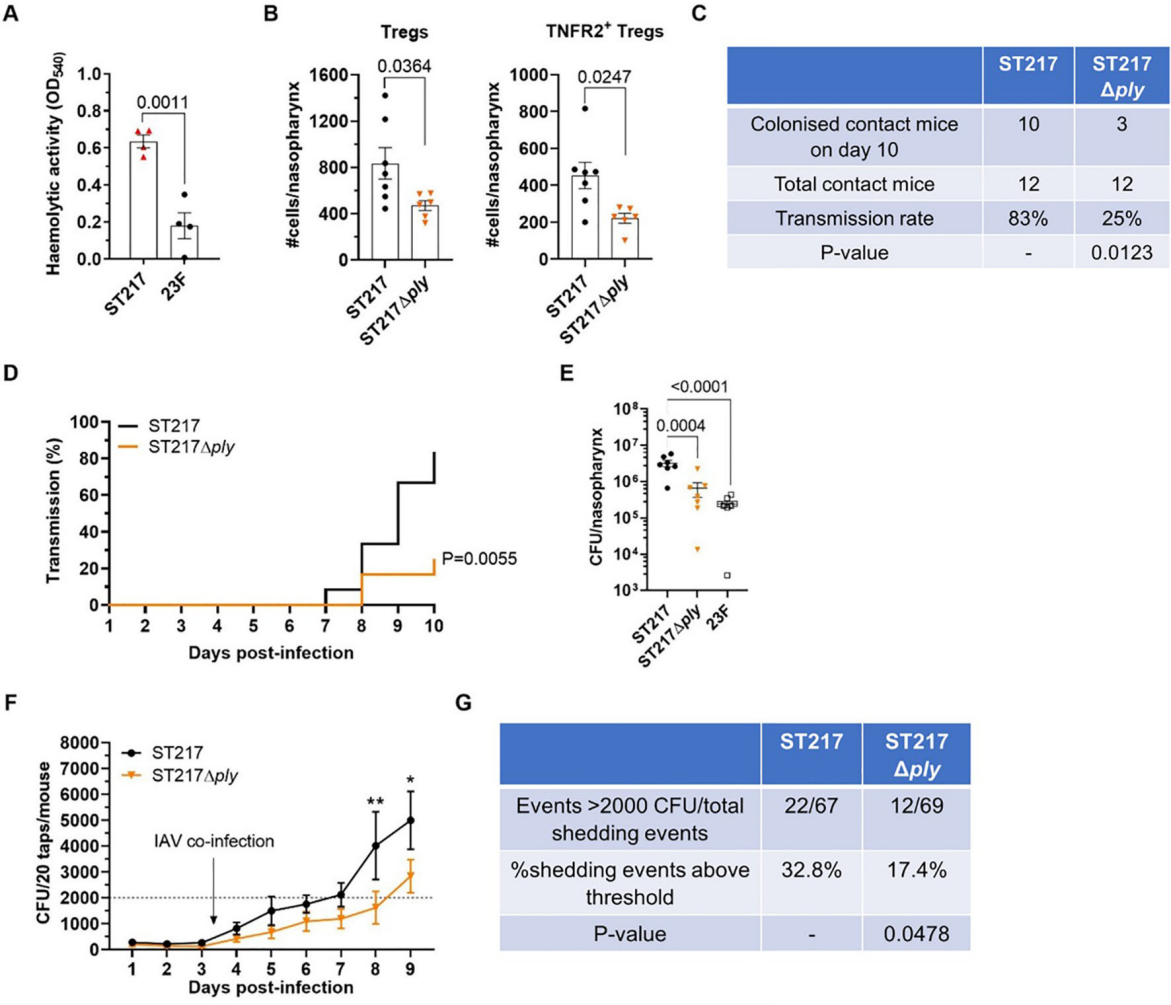
Treg activation and pneumococcal transmission is dependent on pneumolysin. **(A)** Haemolytic activity of ST217 and 23F. Shown is the average level (± SEM) of haemoglobin released (OD_540_) following incubation with red blood cells. Data shown are from four independent experiments. Groups were compared with unpaired t-test. **(B)** Number of (TNFR2^+^) Tregs (CD3^+^CD4^+^Foxp3^+^) in mice infected with ST217 (n=7) and ST217Δ*ply* (n=6). Data were from two independent experiments and analysed with an unpaired t-test. **(C)** Number of colonised contact mice and transmission rate (%) on day 10 post-infection. Transmission was monitored in 4 cages (n=12 contact mice) in two independent experiments. Transmission rates were compared with a Fisher’s exact test. **(D)** Transmission rate over time of ST217 and ST217Δ*ply*. Data shown are the percentages of colonised contact mice (n=12) per day. Contact mice were considered colonised if pneumococci could be recovered on two consecutive days by nose tapping. Data were analysed with a Mantel-Cox test. **(E)** Pneumococcal density (± SEM) on day 10 of carriage for ST217 (n=7), ST217Δ*ply* (n=7), and 23F (n=8) following IAV co-infection. Groups were compared with ordinary one-way ANOVA followed by Holm-Sidak’s multiple comparisons test. Data were from two independent experiments. **(F)** Daily average pneumococcal shedding levels (CFU/20 nose taps/mice [± SEM]) of ST217 and ST217Δ*ply* (n=8 per group). Dashed line shows the transmission threshold of 2000 CFU/20 nose taps. Shedding over time was analysed with multiple t-tests (corrected with Holm-Sidak method). **(G)** Percentage of shedding events that exceed the transmission threshold of 2000 CFU/20 nose taps. Data were analysed with a Fisher’s exact test. *P<0.05. **P<0.01. IAV, Influenza A virus.

Previous studies have shown that Ply is essential for inducing Treg responses during carriage and for establishing carriage and prolonging carriage duration (11, 25). While Ply is expressed by almost all strains, its sequence can vary and can exhibit diverse antigenic properties (26, 32). To explore the role of Ply in Treg responses and transmission, we first assessed the haemolytic activity of Ply from ST217 and 23F. Strikingly, ST217 displayed significantly higher haemolytic activity than 23F, indicating a link between Ply activity and transmission potential (**Figure 6A**). Genome sequencing showed that Ply of 23F exhibited one non-synonymous single nucleotide polymorphism at amino acid position 380, resulting in a substitution from Aspartic Acid (D) to Asparagine (N) compared to ST217. This mutation is located within the membrane-binding region of pneumolysin (domain 4), which could explain the differences in haemolytic activity between strains (33) (**Supplementary Figure 8**).

We then compared the carriage and transmission dynamics of ST217 and its Ply-knockout mutant (ST217Δ*ply*). Mice infected with ST217Δ*ply* displayed a lower (TNFR2^+^) Treg response on day 2 p.i. (**Figure 6B**), correlating with delayed and reduced transmission (25% KO vs 83% WT) (**Figures 6C, D**). This lower TNFR2^+^ Treg response is due to a reduction in total Treg numbers, as the proportion of TNFR2^+^ Tregs is similar between WT and KO strains (**Supplementary Figure 6**). Ply-deficiency also reduced the carriage density of ST217 to levels similar to the low-transmissible serotype 23F, indicating that Ply is essential for maintaining high carriage density (**Figure 6E**). This reduced density was associated with lower shedding levels (P = 0.0088 day 8; P = 0.0225 day 9) and fewer shedding events exceeding the transmission threshold (17.4% for KO vs 32.8% for WT) (**Figures 6F, G**). These findings demonstrate that Ply is essential for inducing TNFR2^+^ Tregs responses and maintaining the high carriage density of ST217, thereby increasing shedding and promoting transmission.

## Discussion

Hypervirulent serotype 1 (ST217) is the main cause of pneumococcal disease outbreaks in Sub-Saharan Africa (3). This study describes the important role of TNFR2^+^ Tregs in host-to-host ST217 transmission. Activation of TNFR2^+^ Tregs is key to attenuating the inflammatory response by suppressing (IL-17A-producing) γδT17 and Th17 cells, leading to reduced neutrophil and macrophage responses in the nasopharynx during carriage. However, the ability to downregulate inflammation and suppress cellular immune responses has the unwanted consequence of increasing nasopharyngeal colonisation density of ST217, thereby enhancing shedding to levels that allow transmission to new hosts. Paradoxically, therefore, suppressive TNFR2^+^ Tregs create the necessary conditions to promote increased ST217 transmission. This is not the case for serotype 23F however where TNFR2^+^ Tregs are significantly lower, with neutrophil and macrophage numbers significantly higher as a consequence, leading to lower nasopharyngeal pneumococcal density and significantly reduced transmission. Hence, the ability to drive TNFR2+ Treg responses does not appear to be uniformly consistent across different pneumococcal serotypes, which intriguingly may explain why some serotypes are significantly more invasive than others.

In order to further define this difference, we investigated carriage density and found that the highly transmissible ST217 strain carried at a higher nasopharyngeal density compared to 23F. Higher carriage densities increase the risk of onward transmission as they increase the number of pneumococci exiting a host (27). Indeed, our results show that ST217 displays significantly higher shedding levels than 23F. This is consistent with previous research showing that increased colonisation density correlates with increased transmission potential (27, 34). These studies, along with our findings, suggest that a high colonisation density is a hallmark of a highly transmissible strain. Increased colonisation densities are also a known risk factor for pneumococcal disease, linking disease risk to transmission potential (35, 36). Therefore, identifying methods to reduce carriage density could be essential for lowering disease risk and disrupting transmission. This is particularly important in high-disease burden areas like Sub-Saharan Africa, where factors such as air pollution and co-infection with soil-transmitted helminths or HIV promote pneumococcal carriage (37–39).

Our data demonstrate that during ST217 colonisation, Tregs maintain high carriage density, significantly contributing to pneumococcal transmission. This Treg-mediated control of carriage and subsequent transmission depends on TNFR2 signalling, as TNFR2^-/-^ mice display diminished Treg responses in the nasopharynx, leading to lower pneumococcal densities and reduced shedding and transmission. While our study shows that TNFR2^+^ Tregs promote transmission, we have previously demonstrated that these cells also play a crucial role in protection against fatal bacteraemia during pneumococcal pneumonia (6). By suppressing excessive inflammation, Tregs reduce damage to the airway epithelium, thereby preventing *S. pneumoniae* from accessing underlying tissues and the bloodstream (6). Thus, targeting Tregs may disrupt transmission in highly virulent strains, but could also potentially lead to susceptibility to invasive pneumococcal disease. This ‘double-edged sword’ effect, where Tregs protect against invasive disease in the lower respiratory tract while maintaining bacterial persistence in the upper respiratory tract, is at the core of our understanding of niche-specific host immunity to pneumococcal infection.

While TNFR2^-/-^ mice display significantly impaired Treg responses and are phenotypically similar to Treg-depleted mice, we acknowledge that the contribution from other TNFR2^+^ cell populations cannot be excluded. Although TNFR2 is preferentially expressed by Tregs, effector T-cells can also upregulate TNFR2 following activation (40). Additionally, TNFR2 signalling has been shown to influence macrophage polarisation, with TNFR2 stimulation driving macrophages toward an anti-inflammatory M2-like phenotype, which may contribute to controlling inflammation (41). Further studies are therefore required to elucidate the relative roles of Tregs and other TNFR2^+^ populations in controlling inflammation during pneumococcal infection.

Based on our data, IL-17A-producing immune cells, such as Th17 cells, play an essential role in impairing pneumococcal shedding and transmission by enhancing neutrophil-mediated clearance of *S. pneumoniae* (7, 8). We observed high IL-17A levels and increased neutrophil-mediated pneumococcal clearance during early carriage of low-transmission serotype 23F. Additionally, Treg-mediated suppression of neutrophil recruitment promotes pneumococcal persistence at high densities in the nasopharynx, thereby increasing shedding and transmission. This is in agreement with previous studies demonstrating that neutrophil depletion or inactivation increases pneumococcal load in the nasopharynx and enhances transmission (34, 42).

We observed a robust γδT17 response and high IL-17A levels on day 3 p.i., suggesting that γδT17 cells are a principal source of IL-17A during pneumococcal colonisation. Treg-mediated suppression of γδT17 cells during early carriage is essential for enabling high shedding, as evidenced by the heightened γδT17 response and diminished shedding in DEREG mice. Additionally, γδT17 responses increase and persist throughout pneumococcal carriage, suggesting their role in later stages of infection. This observation is consistent with findings from other infections, such as *Mycobacterium tuberculosis*, where γδT17 cells, rather than Th17 cells, are the main source of IL-17A, even at 52 weeks p.i. (43). Our results demonstrate that γδT17 cells, in addition to Th17 cells, play a key role in controlling pneumococcal carriage and transmission. The concept that IL-17A-producing cells may protect against pneumococcal transmission is supported by previous studies (44, 45). For instance, low IL-17A levels were measured in children with high pneumococcal density in the nasopharynx, which could indicate increased shedding and transmission potential (44). Moreover, children with a polymorphism in the promoter region of IL-17A have a significantly increased risk of pneumococcal colonisation, suggesting IL-17A is key for protection against *S. pneumoniae* acquisition (45). The role of IL-17A-mediated inflammation in pneumococcal colonisation and shedding merits more investigation and could be a key focus for vaccine research aimed at disrupting transmission.

We demonstrate that TNFR2^+^ Tregs are drivers of transmission and that their activation is strain-dependent, which may explain the strain-to-strain differences in shedding and transmission observed in this study and others (18, 27). Highly transmissible strains like ST217 elicit strong (TNFR2^+^) Treg responses, correlating with increased carriage density and shedding. We show that Treg activation is dependent on Ply, a highly conserved virulence factor important for establishing and maintaining carriage (11, 25). Strains like ST217 with potent Ply, exhibit higher pneumococcal densities, shedding levels, and transmission rates compared to strains with low Ply activity, such as 23F. This suggests that Ply-mediated Treg induction is one of the mechanisms exploited by *S. pneumoniae* to persist in the nasopharynx and drive transmission. Potential mechanisms behind Ply-driven induction of Tregs include Ply-mediated polarisation of naïve CD4^+^ T-cells into Foxp3^+^ Tregs through interaction with Mannose-Receptor C type 1 on dendritic cells (46) and Ply-induced TGF-β1 responses in nasopharyngeal fibroblasts and epithelial cells, with TGF-β1 being an essential trigger for the induction of Treg responses during pneumococcal carriage (11).

Collectively, our findings provide interesting insight into the immunological factors that affect pneumococcal carriage and transmission. We demonstrate that Tregs contribute significantly to the chronicity of bacterial infections and somewhat unexpectedly facilitate their onward transmission to new hosts. Unravelling the molecular pathways that activate Tregs during bacterial infection and further investigation of the cellular targets of Tregs are needed to fully understand the interplay between respiratory pathogens and the host, and importantly, novel Treg modulatory therapies to control bacterial shedding and transmission.

## Data Availability

The original contributions presented in the study are included in the article/**Supplementary Material**. Further inquiries can be directed to the corresponding author.
