# Supplementary material for: Pneumococcal transmission is driven by TNFR2+ regulatory T-cells
Source: Front Immunol. 2026 Jul 20;17:1840694. doi: 10.3389/fimmu.2026.1840694 (PMC13429388; doi:10.3389/fimmu.2026.1840694)
Supplement: Supplementary file 1 [file DataSheet1.docx]

## **Supplementary files**


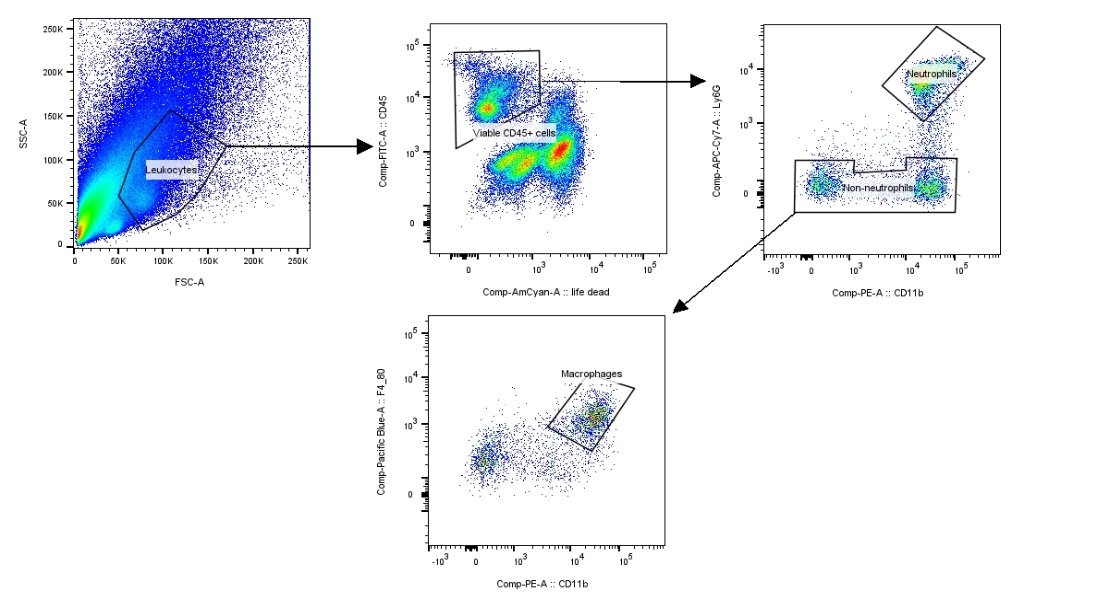

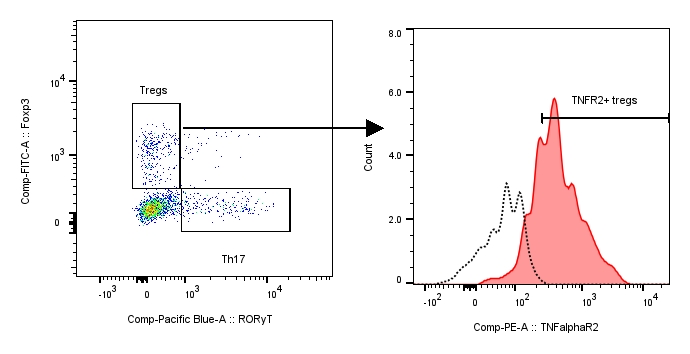

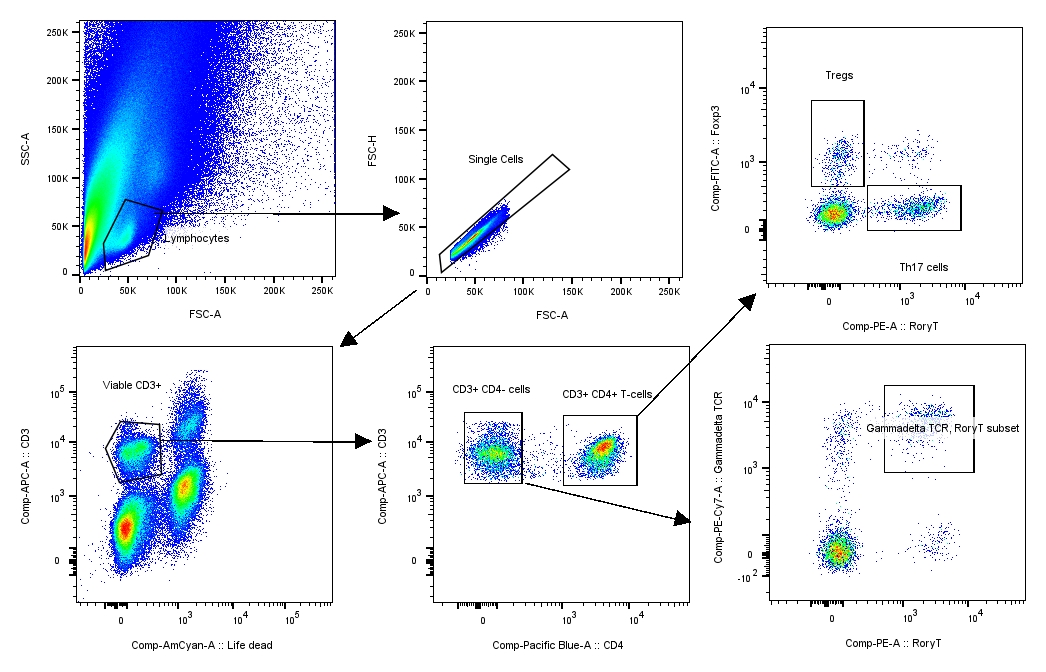


**A**

**B**

**C**

**Supplementary figure 1. Gating strategies of flow cytometry.** Representative flow cytometry plots to show how the number of Tregs (CD3^+^CD4^+^Foxp3^+^), Th17 cells (CD3^+^CD4^+^RORγt^+^), γδT-cells (CD3^+^CD4^-^γδTCR^+^), and γδT17-cells (CD3^+^CD4^-^γδTCR^+^RORγt^+^) (Antibody panel 1; **A**) and neutrophils (Ly6G^+^CD11b^+^) and macrophages (F4/80^+^ CD11b^+^) (Antibody panel 2; **B**) are quantified in a nasopharyngeal sample. Tregs were further characterised as TNFR2^+^ and TNFR2^-^ (**C**). Shown is the TNFR2^+^ Treg population in WT C57BL/6J (red) and TNFR2^-/-^ mice control (black dotted line).

**Supplementary Table 1. Antibody panels used to stain nasopharyngeal cell suspensions.**

| Panel 1 | **Antibody** | **Source** |
| --- | --- | --- |
|  | CD3-Allophycocyanin (APC) | Biolegend, London, UK |
|  | CD4-APC/Cyanine7 | Biolegend |
|  | TNFR type II-Phycoerythrin (PE) | Biolegend |
|  | TCR γ/δ-PE/Cyanine7 | Biolegend |
|  | Foxp3-Alexa fluor488 | Biolegend |
|  | RORγt-BV421 | BD Horizon^TM^, Wokingham, UK |
| Panel 2 | **Antibody** | **Source** |
|  | CD3-APC | Biolegend |
|  | CD4-VioBlue® | Miltenyi Biotec, Bisley, UK |
|  | TCR γ/δ-PE/Cyanine7 | Biolegend |
|  | Foxp3-Alexa fluor488 | Biolegend |
|  | RORγt-PE | BD Horizon^TM^ |
| Panel 3 | **Antibody** | **Source** |
|  | CD45-Fluorescein isothiocyanate (FITC) | Biolegend |
|  | Ly6G-APC/Cyanine7 | Biolegend |
|  | CD11b-PE | Biolegend |
|  | F4/80-Pacific Blue | Biolegend |

**
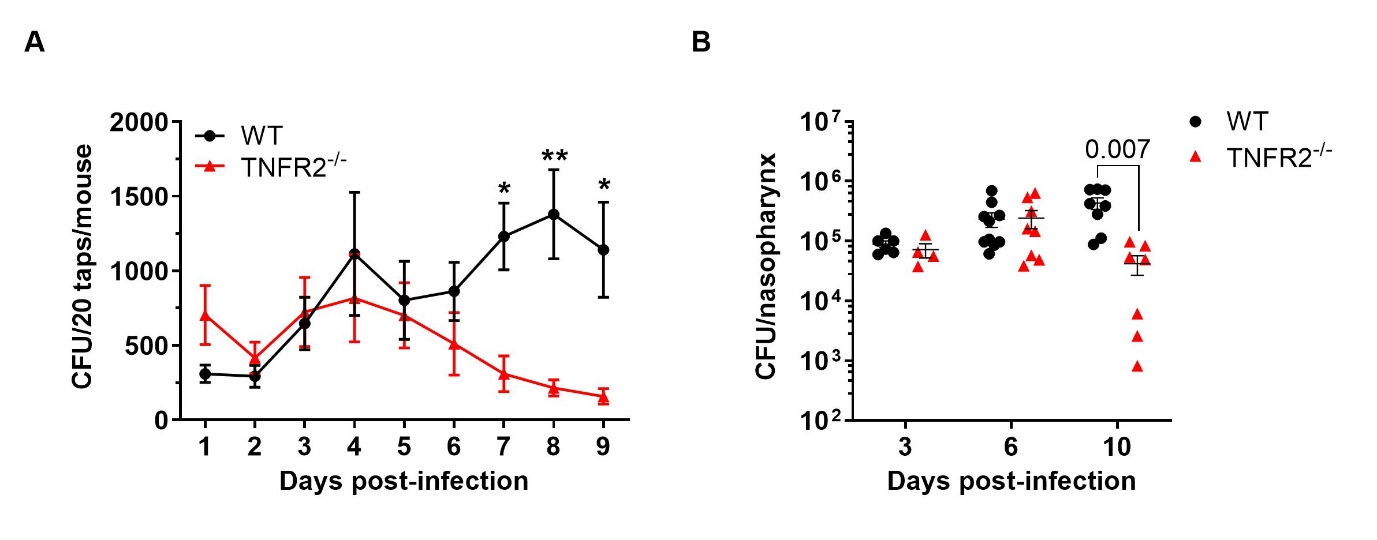
Supplementary figure 2. Shedding levels and pneumococcal density in WT and TNFR2^-/-^** **mice in the absence of IAV**. (**A-B**) WT and TNFR2^-/-^ C57BL/6J mice were intranasally infected with ST217. (**A**) Daily average shedding levels of ST217 (CFU/20 nose taps/mice [± SEM]) in WT (n=9) and TNFR2^-/-^ (n=10) mice. (**B**) Pneumococcal density (± SEM) in the nasopharynx of WT and TNFR2^-/-^ mice. Carriage densities and shedding levels were compared with Multiple t-tests (corrected with Holm-Sidak method). *P<0.05. **P<0.01. IAV: Influenza A virus.


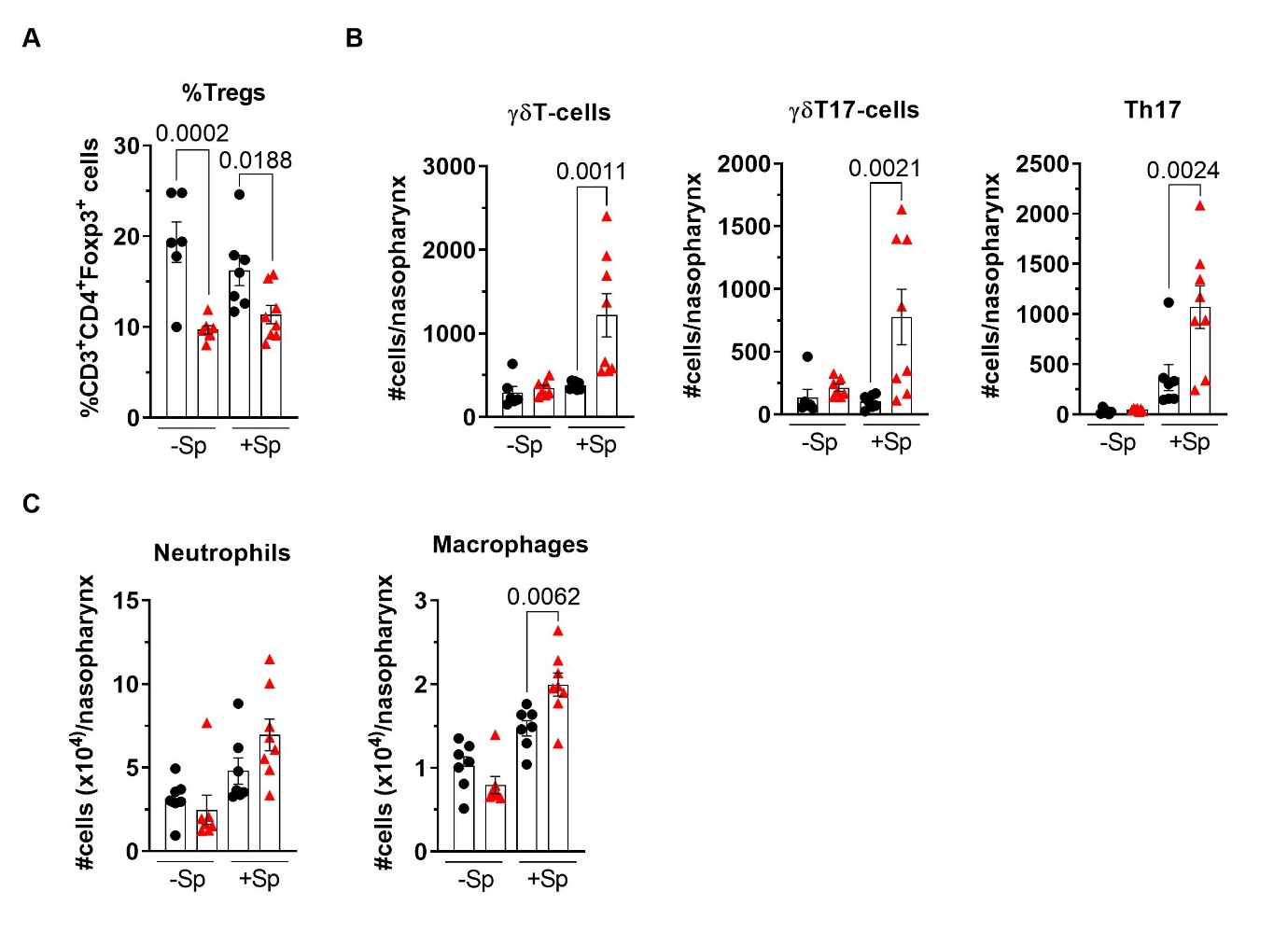
**Supplementary figure 3. Immune cell responses in WT and TNFR2^-/-^ mice in the absence of IAV.** Female WT and TNFR2**^-/-^** C57BL/6J were intranasally infected with ST217 (+Sp). Immune responses were determined in the nasopharynx on day 6 post-infection using flow cytometry. Naïve mice acted as controls (-Sp). (**A**) Treg (CD3^+^CD4^+^Foxp3^+^) proportions in the nasopharynx of WT and TNFR2^-/-^ mice. (**B**) Number of γδT-cells (CD3^+^CD4^-^γδTCR^+^), γδT17-cells (CD3^+^CD4^-^γδTCR^+^RORγt^+^), and Th17 cells (CD3^+^CD4^+^RORγt^+^) in the nasopharynx of WT and TNFR2^-/-^ mice. (**C**) Number of neutrophils (Ly6G^+^CD11b^+^) and macrophages (CD11b^+^, F4/80^+^) in the nasopharynx of WT and TNFR2^-/-^ mice. Data was analysed with ordinary one-way ANOVA with Holm-Sidak’s multiple comparisons test. IAV: Influenza A virus. Sp: *S. pneumoniae*.


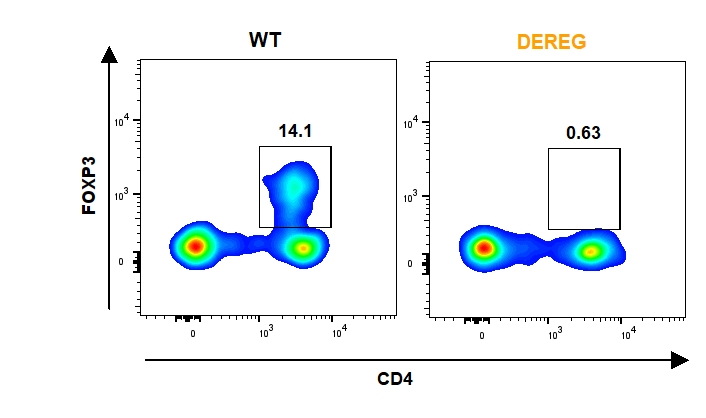


**Supplementary figure 4. Treg depletion in DEREG mice.** DEREG and WT mice were intraperitoneally treated with diphtheria toxin (1µg in 100 µl PBS) on days 0 and 1 of carriage. Data shown are representative flow plots of the frequency of Tregs (CD3^+^CD4^+^Foxp3^+^) in WT and DEREG mice.


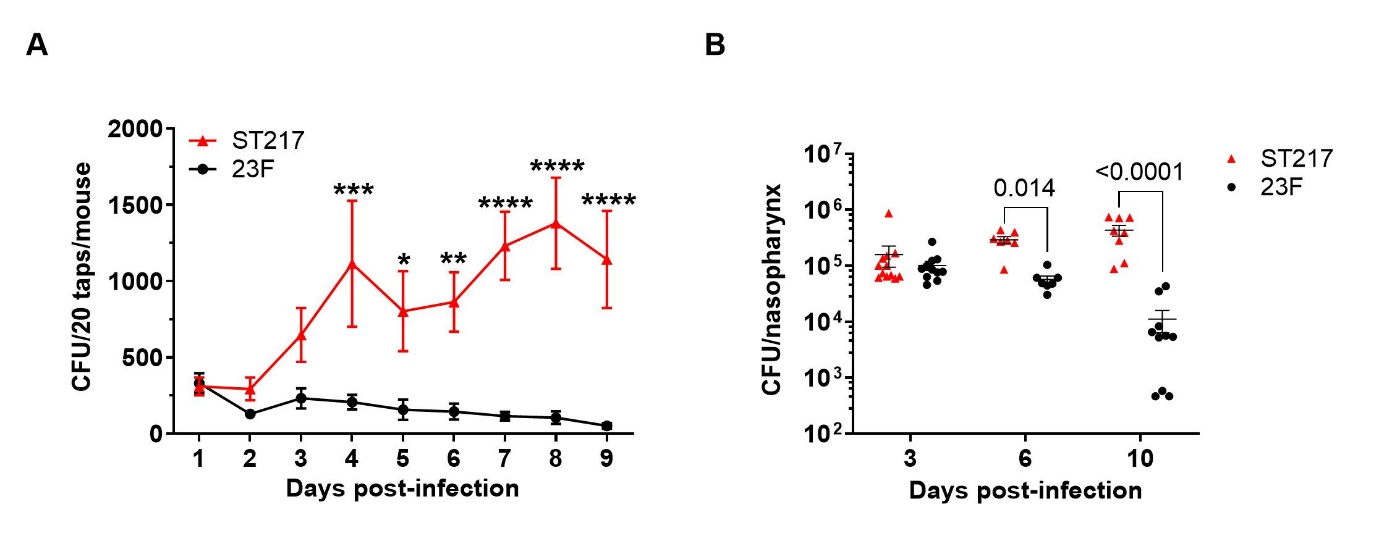


**Supplementary figure 5. Shedding and pneumococcal density of ST217 and 23F in the absence of IAV.** (**A**) Daily average pneumococcal shedding levels (CFU/20 nose taps/mice [± SEM]) of ST217 (n=9) and 23F (n=10). (**B**) Pneumococcal density in the nasopharynx of ST217- and 23F-infected mice. Shedding levels and pneumococcal densities between strains were compared with multiple t-tests (corrected with Holm-Sidak method). *P<0.05. **P<0.01. ***P<0.001. ****P<0.0001. IAV: Influenza A virus.

**
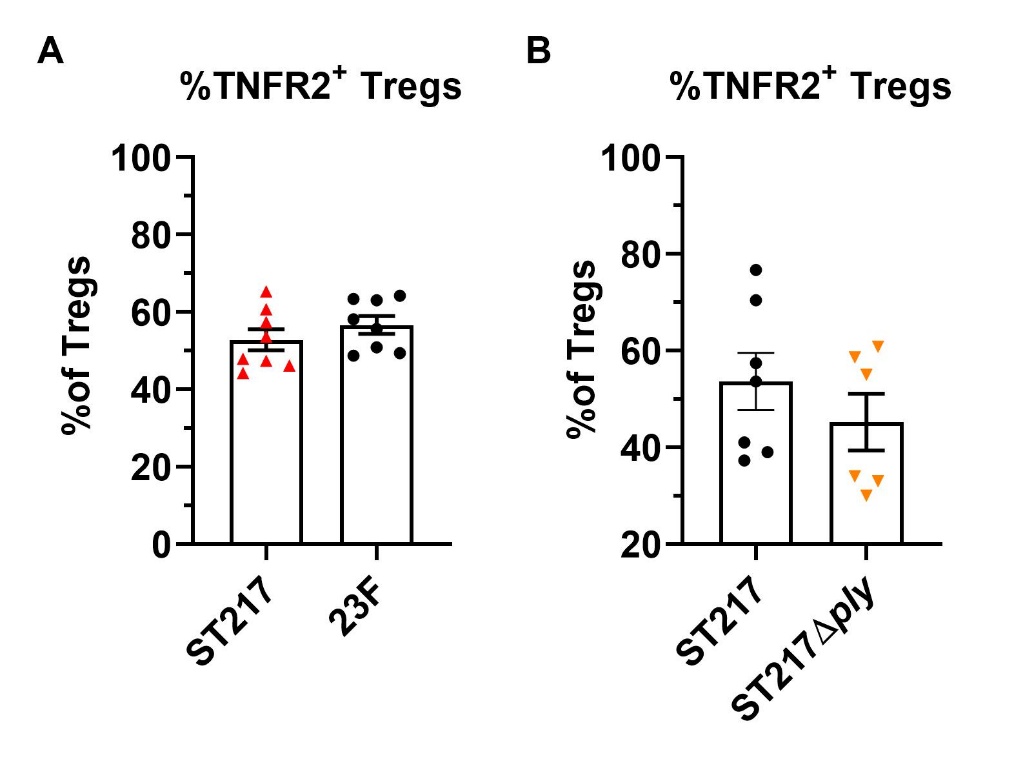
Supplementary figure 6. ST217-, ST217Δ*ply*- and 23F-infected mice display similar proportions of TNFR2^+^ Tregs.** Female C57BL/6J mice were intranasally infected with a carriage dose of ST217, ST217Δ*ply*, or 23F. Flow cytometry was used to determine immune responses in the nasopharynx on day 2 post-infection. (**A**) The proportion of TNFR2^+^ cells within total Tregs (CD3^+^CD4^+^Foxp3^+^) between ST217 (n=8) and 23F (n=8). Data shown are from three independent experiments. (**B**) The proportion of TNFR2^+^ cells within total Tregs between ST217 (n=7) and ST217Δ*ply* (n=6). Data were from two independent experiments. Groups were compared with unpaired t-tests.

**
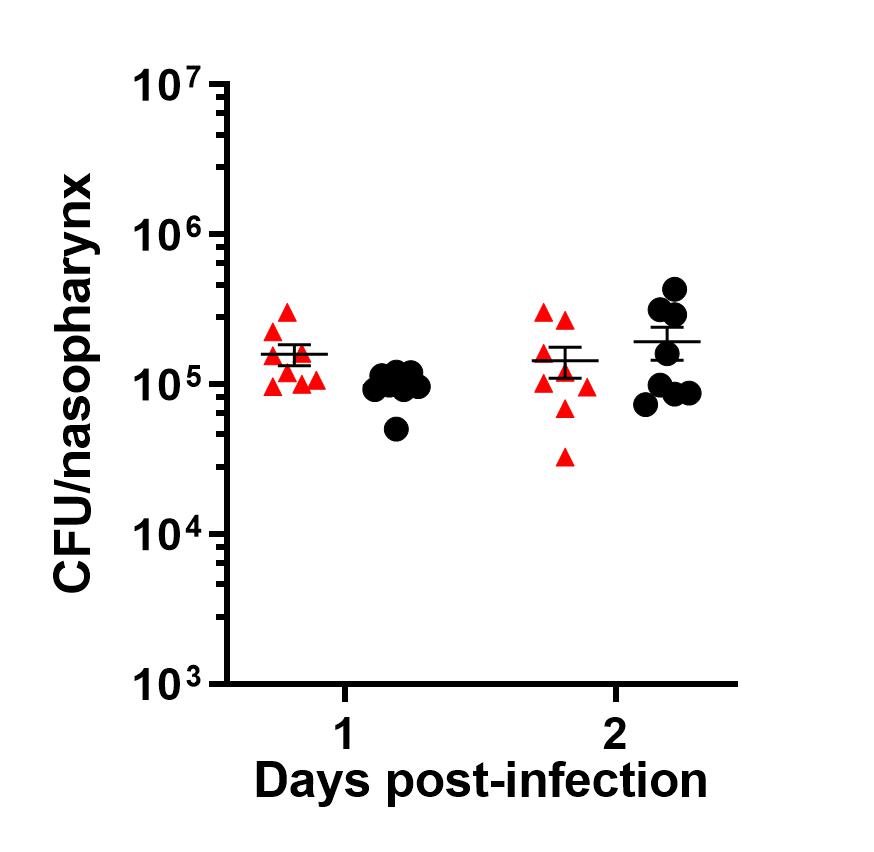
**

**Supplementary figure 7. Pneumococcal colonisation density during early stages of carriage.** Female C57BL/6J mice were intranasally infected with pneumococcal serotype 1 (ST217) or 23F. Data shown is the pneumococcal density in the nasopharynx of ST217- and 23F-infected mice on days 1 and 2 of carriage.

*
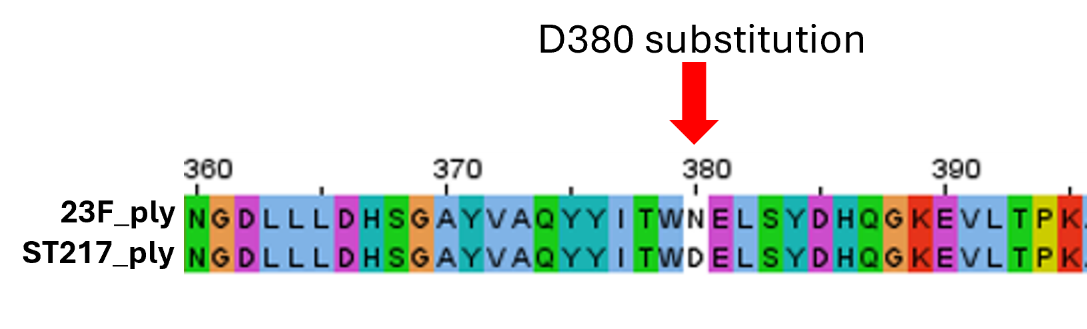
*

**23F**

**ST217**


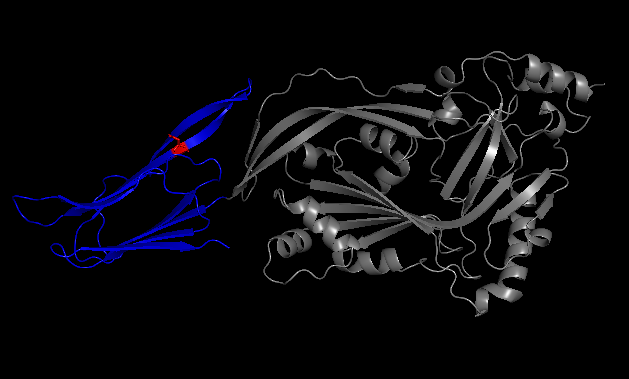

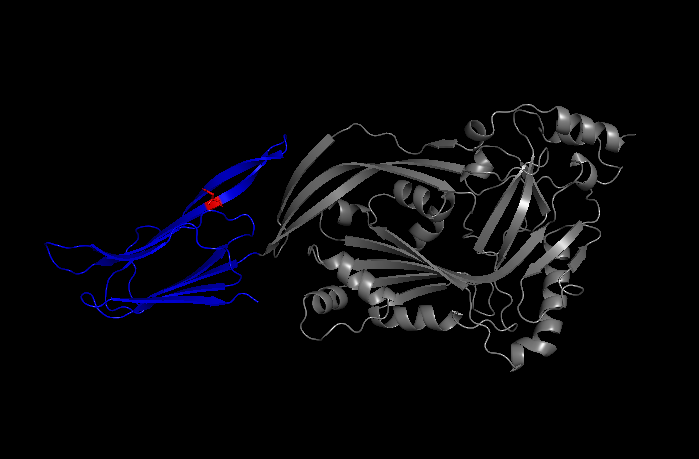


**B**

**A**

**Supplementary figure 8. Pneumolysin sequence** **and predicted protein structure**. Shown are the amino acid sequences and the substitution at location 380 in 23F (**A**) and the alpha-fold structures visualised in PyMOL with domain 4 of pneumolysin (blue) and amino acid 380 (red) for ST217 and 23F (**B**).
